# Understanding Social Communication Differences in Autism Spectrum Disorder and First-Degree Relatives: A Study of Looking and Speaking

**DOI:** 10.1007/s10803-019-03969-3

**Published:** 2019-03-12

**Authors:** Michelle Lee, Kritika Nayar, Nell Maltman, Daniel Hamburger, Gary E. Martin, Peter C. Gordon, Molly Losh

**Affiliations:** 1 Northwestern University, 2240 Campus Drive, Frances Searle Building, Evanston, IL 60208 USA; 2 University of North Carolina at Chapel Hill, Chapel Hill, NC USA; 3 St. John’s University, New York, NY USA

**Keywords:** Autism spectrum disorder, Language, Communication, Narrative, Eye gaze, Visual attention

## Abstract

This study examined narrative ability in ASD and parents across two contexts differing in structure and emotional content, and explored gaze patterns that may underlie narrative differences by presenting narrative tasks on an eye tracker. Participants included 37 individuals with ASD and 38 controls, 151 parents of individuals with ASD and 63 parent controls. The ASD and ASD parent groups demonstrated lower narrative quality than controls in the less structured narrative task only. Subtler, context-dependent differences emerged in gaze and showed some associations with narrative quality. Results indicate a narrative ability profile that may reflect genetic liability to ASD, and subtle links between visual attention and complex language skills that may be influenced by ASD genetic risk.

## Introduction

Impairments in social communication constitute a defining feature of autism spectrum disorder (ASD) that can seriously impact competence across social contexts (Tager-Flusberg 2000). For instance, a number of studies have documented impoverished narrative skills in ASD, particularly in unstructured and emotionally salient contexts (e.g., conversational narrative) that are strongly reliant on social cognitive abilities, such as reading thoughts and emotions of protagonists or conversational partners (e.g., Losh and Capps 2003, 2006). In line with this observation, a number of studies have documented links between narrative impairments in ASD and deficits in social cognitive skills, where difficulty reading others’ emotions and cognitive states can limit the ability to build meaningfully on narrative topics and evaluate a communicative partner’s understanding and engagement (Capps et al. 1998; Losh and Capps 2003; Ochs and Capps 2001; Tager-Flusberg and Sullivan 1995).

Subclinical differences in narrative (and broader pragmatic skills) have also been documented among parents of individuals with ASD, and are considered a core feature of the broad autism phenotype (BAP) (Landa et al. 1991, 1992; Piven et al. 1997; Losh et al. 2008, 2012). The BAP refers to subclinical personality and language traits observed at elevated rates among parents of individuals with ASD that are believed to reflect genetic liability to ASD (Piven et al. 1997; Bernier et al. 2012; Bolton et al. 1994; Losh et al. 2008; Virkud et al. 2009). In a landmark study aimed at further defining the language characteristics of the BAP, Landa and colleagues reported evidence of impoverished narrative skills in parents of individuals with ASD, relative to parents of individuals with Down syndrome included as a control for the influence of parenting a child with a developmental disability (Landa et al. 1991). Specifically, parents of individuals with ASD produced narratives that were lower in complexity and coherence than those of controls. These patterns have been mirrored in studies of conversational discourse of parents of individuals with ASD, which have noted increased tangential language and less contingent conversational contributions (Landa et al. 1992; Losh et al. 2008, 2012; Piven et al. 1997). Considered with the extensive literature documenting narrative impairments in ASD, these findings suggest qualitatively similar narrative differences in parents, implicating narrative as a skill potentially impacted by genetic liability to ASD.

An important next step in evaluating the significance of narrative as a trait influenced by genetic liability to ASD will be to understand whether there exist similar profiles of strength and weakness across structured and unstructured contexts in both ASD and among first-degree relatives. Furthermore, exploring potentially underpinning processes related to narrative deficits and differences in ASD and the BAP is critical for understanding whether common underlying mechanisms contribute to observed narrative profiles. As noted previously, social cognition appears to importantly relate to the narrative impairments in ASD, perhaps implicating differences in social attention and perception as important sources of narrative differences. Attention to less socially salient aspects of a scene, for instance, could impact the ability to formulate narratives around meaningful themes and infer motivations of protagonists to build coherent stories. Although this question has not been directly addressed in the BAP, differences in social cognition have been reported in parents (Adolphs et al. 2008; Losh et al. 2009; Losh and Piven 2007; Baron-Cohen and Hammer 1997), and in one study were linked to differences in parents’ pragmatic skills in conversation (Losh and Piven 2007). Identification of such features impacted in both ASD and the BAP, and linked with broader language and related phenotypes associated with ASD and the BAP, might provide a window into those core skills impacted by ASD genetic risk and their neuropsychological origins.

Analysis of eye gaze may provide such an intermediate link, with potential to reveal attentional and perceptual differences that stem from underlying neurobiological variation influenced by ASD genetic risk, and that impact clinical-behavioral phenomena such as narrative and social behavior (Klin et al. 2002). Differences in visual attention have been repeatedly documented in ASD (see Chita-Tegmark 2016; Frazier et al. 2017; Papagiannopoulou et al. 2014 for reviews), and attentional differences during dynamic social scenes have been found to predict greater language and social-communicative impairment in individuals with ASD (Flavell et al. 1981; Jones et al. 2008; Klin et al. 2002; Righi et al. 2018; Speer et al. 2007). Studies of parents of individuals with ASD have also shown differences in visual attention to social scenes. Groen et al. (2012) reported reduced visual attention to socially relevant aspects of brief videos in both parents and their children with ASD. In a study of face processing in the BAP, Adolphs et al. (2008) found that when determining affective expressions of faces, parents of individuals with ASD who displayed the BAP showed a marked reduction in reliance on the eye region, along with increased utilization of the mouth region, relative to controls and parents without the BAP (a pattern that paralleled patterns observed in ASD; Spezio et al. 2007). Considering findings that visual attention patterns appear highly heritable in twins (Constantino et al. 2017), studies of gaze in ASD and unaffected first-degree relatives may serve as a promising avenue for identifying neurocognitive features related to complex behavioral phenotypes, as well as a quantifiable target to utilize in studies of phenotypes linked to molecular-genetic variation in ASD.

In this study, we explored the relationship between visual attention and narrative ability in both ASD and in parents of individuals with ASD. Our objectives were twofold—first, we aimed to document the narrative profiles in ASD and among parents across discourse contexts that differed in structure and emotional content (shown to be critical in revealing broader pragmatic impairments in ASD). In line with prior studies of narrative in ASD (Losh and Capps 2003, 2006; Tager-Flusberg and Sullivan 1995; Losh and Gordon 2014) we predicted that both the ASD and ASD parent groups would show greater differences from controls in the less structured, more emotionally evocative context. Second, we explored potential links between narrative profiles and patterns of visual attention in these same participants. For this exploratory aim, we presented narrative stimuli on an eye tracker, and characterized gaze patterns in relationship to narrative quality. Although predictions were less clear for these data given the lack of prior research examining the relationship between narrative quality and gaze in general, and among individuals with ASD in particular, we hypothesized that previously reported differences in narrative quality in ASD and among parents might stem from underlying differences in visual attention. As such, we predicted that lower quality narrative would be related to decreased attention to social images during narration.

## Methods

### Participants

Participants included 37 individuals with ASD and 38 typically developing controls without a family history of ASD, as well as 151 parents of individuals with ASD and 63 parent controls without a personal or family history of ASD. All participants spoke English as their first language. Participants were recruited through registries and local resources in the Midwestern United States (e.g., autism advocacy groups, area education agencies, health clinics, recruitment at community events, etc). Participants were excluded if they reported a family history of genetic disorders related to ASD (e.g., fragile X syndrome, tuberous sclerosis). Informed consent was appropriately obtained and all procedures were approved by the University Institutional Review Board.

Demographic information is presented in Table 1. Verbal IQ (VIQ) and Performance IQ (PIQ) were assessed with the Wechsler Abbreviated Scale of Intelligence (WASI) or the Wechsler Adult Intelligence Scale (WAIS)—Third or Fourth Editions (Wechsler 1997, 1999, 2008). Inclusion criteria for participants with ASD and controls included being 15 years of age and older, and having a Full Scale IQ (FSIQ) and Verbal IQ (VIQ) ≥ 80. Diagnostic status was confirmed by administration of the Autism Diagnostic Observation Schedule-Second Edition (ADOS-2) (Lord et al. 2012) and/or Autism Diagnostic Interview-Revised (ADI-R) (Lord et al. 1994), and evaluation of whether symptoms were consistent with DSM-IV (APA 1994) or, once published, DSM-5 (APA 2013) criteria. Parents of individuals with ASD had at least one child with a confirmed diagnosis of ASD. Every effort was made to recruit intact families (e.g., parent–child dyads); however, in some cases the individual with ASD did not qualify for the present study due to IQ limitations, or other exclusion criteria, or parents were not available. This resulted in 49 total parent–child dyads in the ASD group.

**Table 1:** Demographic information

	ASD group	ASD control group	ASD parent group	Parent control group
	(n = 37)	(n = 38)	(n = 151)	(n = 63)
	M (SD)	M (SD)	M (SD)	M (SD)
FSIQ (SD)	106.41 (13.70)*	117.55 (11.85)	112.00 (10.81)*	115.76 (10.05)
VIQ (SD)	105.27 (13.68)*	119.55 (11.67)	110.38 (11.08)	112.49 (11.49)
PIQ (SD)	106.46 (15.57)	111.83 (12.43)	110.95 (11.17)	115.00 (11.73)
Age (SD)	23.02 (7.77)	20.74 (4.83)	45.99 (7.32)*	41.85 (10.18)
Sex (M:F)	27:10	17:21	64:87	25:38

ASD, autism spectrum disorder; M, mean, SD, standard deviation

*p < 0.05

### Assessment of the Broad Autism Phenotype (BAP) in Parents

The Modified Personality Assessment Scale (MPAS-R) is a standardized personality interview that has been used to define personality features of the BAP (Losh et al. 2008; Piven et al. 1994, 1997). The MPAS-R consists of a series of open-ended questions that probe for different personality traits associated with the BAP (e.g., social reticence) that are rated by examiners blind to group, according to established procedures (see Losh et al. 2008; Piven et al. 1994, 1997). Questions probe for both trait endorsement as well as concrete behavioral examples to substantiate endorsements. Following prior work (Losh et al. 2008; Piven et al. 1994, 1997), the presence of each trait was rated on a three-point scale ranging from 0 to 2, with 2 representing definite presence of the trait, 0 as absent, and 1 as not clearly present (0 and 1 conservatively collapsed as “absent” for BAP group assignment). All interviews were videotaped and independently rated by two coders blind to group membership. BAP status was determined through consensus discussion for the presence of each trait based on self-report. Intra-class correlations were calculated for each BAP trait prior to consensus: ICC (1.8); Aloof (0.84), Rigid (0.79), Untactful (0.73). For the purposes of this study, individuals were considered BAP (+) if they received a score of 2 on either social (presenting with “aloof” or “untactful” traits) or rigid scales of the MPAS.

### Narrative Procedures

Narrative tasks were presented on a Tobii T60 series eye tracker. Participants were seated approximately 50–60 cm from the screen. Stimuli were presented on a 17″ TFT monitor with a resolution of 1280 × 1024 pixels.

#### Wordless Picture Book (PB)

*Frog Where Are You?* (Mayer 1969) is a 24-page wordless PB about a boy and his adventures searching for a lost pet frog, and has been used extensively in prior studies of narrative ability (Losh and Capps 2003; Bamberg 1987; Berman and Slobin 1994; Capps et al. 2000; Diehl et al. 2006). This narrative task is considered to be highly structured (given that it contains a series of clearly depicted actions with canonical episodic and plot structure) and has been used extensively in prior studies of narrative ability and development in typical (Bamberg 1987; Berman and Slobin 1994) and atypical populations, including ASD (Capps et al. 2000; Diehl et al. 2006; Losh and Capps 2003). Participants were instructed that they would be telling a story from a wordless picture book. They were then presented with each page, one at a time, and asked to narrate the story while viewing the page. There was no time limit for page presentation, but pages were advanced as participants concluded speaking, consistent with methods employed in prior studies with this narrative task (e.g., Capps et al. 2000; Losh and Capps 2003).

#### Thematic Apperception Test (TAT)

**Fig. 1:**
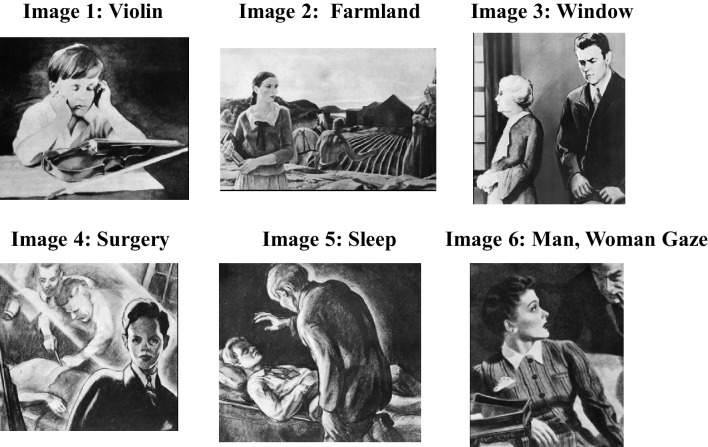
TAT images included

Selected scenes from the TAT (Murray 1943) were utilized to elicit narratives in an open-ended context. The TAT is a projective psychological test that has been applied in studies of narrative (Beaumont and Newcombe 2006; Hiraishi et al. 2012; Lee et al. 2017; Turk et al. 2010). The TAT presents ambiguous and emotionally evocative images from which participants are asked to create narratives. This task was selected for comparison against the more structured wordless picture book, given that individuals with ASD have been shown to exhibit greater difficulty in narrative tasks that are more open-ended and with increased emotional complexity (e.g., Losh and Capps 2003, 2006). Therefore, the TAT served as an excellent task to provide a standardized, yet still open-ended and complex context in which to evaluate narrative and visual attention in these groups. Following prior work utilizing the TAT as a narrative elicitation task (Turk et al. 2010), six unrelated TAT images of varying complexity and emotional content were included (cards 1, 2, 6BM, 8BM, 12M, 13MF; hereafter referred to as Images 1–6 or by a brief descriptor of the image; see Fig. 1 for images). Participants were instructed to tell a story with a beginning, middle, and end, and to include information on what the characters were thinking, feeling, and doing, immediately following the 8-s presentation of each image.

Audio files from all narrative tasks were transcribed by transcribers who were blind to group status and trained to ≥ 80% word reliability for each narrative task. Ten percent of transcripts (across groups and sex) were randomly selected for word reliability assessment in each task (mean = 95%, range = 80–100%).

#### Analysis of Narrative Quality

Narratives were analyzed using Latent Semantic Analysis (LSA; Landauer and Dumais 1997), a computational linguistic tool whereby an individual text sample is automatically compared to other text samples, and which prior work has shown relates to key gold standard hand-coded indices of narrative coherence, such as grammatical complexity and narrative evaluation (see Lee et al. 2017; Losh and Gordon 2014 for a detailed explanation of LSA), which serve essential functions in cohering narrative events and rendering them psychologically and socially meaningful, respectively. For each stimulus or narrative grouping, one control narrative most similar to all other participants was selected as the gold standard and then excluded from group comparisons. A quantitative measure of similarity of each individual narrative to these prototypical narratives, ranging from − 1 to 1 (with 1 indicating perfect similarity), was then generated, and is roughly representative of narrative quality. LSA scores were computed for the PB task in its entirety as well as participants’ narratives in response to the six distinct TAT images separately and averaged across all TAT images.

### Eye Tracking Procedures

Pre-specified areas of interest (AOIs) were applied to each stimulus using Tobii Studio software. AOIs included face and body regions for each character, and nonsocial objects in the images that composed the setting (see Table 2 for summary). Analyses of visual attention patterns used two primary, complementary variables: (1) proportion of looking time to an AOI out of total looking time, and (2) proportion of fixations to an AOI out of total fixations for a stimulus. For the PB, these proportions were calculated by summing the total tracked time or fixations to an area of interest over the total tracked time or fixations across all 24 pages. For the TAT, these variables were calculated for each unique image only.

**Table 2:** Areas of interest (AOI) included for TAT images

Image (title)	AOIs analyzed	Prominent AOI
TAT 1 (violin)	Face	Face
	Body	
TAT 2 (farmland)	Face	Setting
	Body	
	Setting	
TAT 3 (window)	Face	Body
	Body	
	Setting	
TAT 4 (surgery)	Face	Setting
	Body	
	Setting	
TAT 5 (sleep)	Face	Body
	Body	
TAT 6 (man, woman gaze)	Face	Face
	Body	

#### Analysis of Gaze

Gaze was recorded for both eyes during presentation of stimuli, based on a sampling rate of 60 Hz. To account for potential data loss, we defined parameters for fixations consistent with prior research (Wass et al. 2013), who developed parameters to account for possible poor data quality in infant gaze studies. Briefly, these settings reduce the impact of technological error or intra-individual variability (e.g., tendency to move eyes towards the edge of the screen, head movement during tracking tasks, “flickery” gaze data) by defining fixations based on the I-VT fixation filter available in Tobii Studio (Tobii Technology AB, Danderyd, Sweden), including fixation data from both eyes, a velocity threshold of 35°/s, and duration and angle between each new fixation set at 100 ms and 0.5°, respectively. Missing data with gaps no greater than 150 ms were linearly interpolated and a moving average window of 3 samples was used to reduce noise.

Track loss (i.e., time when gaze was not detected by the eye tracker) was necessarily assessed in task-specific ways to account for differences in the nature of the stimuli and administration procedures (e.g., 24 pages of connected narrative stimuli presented continuously with eye movement recording in the PB task vs. 6 distinct images with narrations after viewing the image in the TAT). Because this study was the first to utilize these stimuli on an eye-tracker, concurrent with speaking, there were no set guidelines available for quality control. Rather, for each task, data quality metrics were developed based on detailed analysis of the distribution of tracked time and fixation frequency across participants, in order to assess what might be considered normative track loss for these groups. First, task administrations were reviewed for any factors that may have impacted data quality (e.g., participant distraction) and those data were excluded. Then, the quality of gaze data was additionally assessed as follows.

#### Data Quality Assessment in PB Task

In the PB task, participants were speaking while gaze was recorded across all 24 pages of the story. Given that data quality procedures have not been previously reported for this type of extended eye tracking data during narration, we employed a conservative standard to account for data loss in both spoken words and gaze duration, where participants were excluded if their word-to-tracked eye movement time ratio was greater than 5 words/second during any episode of the PB. Episode definition was informed by prior work, and included the introduction (i.e., setting and instantiation of the “search” theme) the sequence of core search events, and resolution (Losh and Capps 2003; Reilly et al. 1990, 1998; Bamberg and Marchman 1990). Greater than 5 words/second of track time would suggest that gaze was not consistently tracked during a substantial portion of vocalization during that episode. Together, data quality review resulted in the exclusion of 10 (27%) individuals with ASD, 4 (11%) ASD controls, 41 (28%) parents of individuals with ASD, and 13 (21%) parent controls.

For the TAT, where participants narrated their stories after having viewed the scene for 8 s, participants were excluded if their overall fixation count on a given image was < 5 and total fixation duration was < 4 s (i.e., gaze data unreliable for more than half of the 8 s stimulus presentation). These criteria resulted in exclusion of Images 1 (“Violin”) and 3 (“Window”) from group comparison analyses for the ASD and ASD control groups, given that proportions of participants with valid data differed significantly (Image 1 “Violin” 49% ASD vs. 87% control, *z* = 2.94, *p* < 0.01; Image 3 “Window” 54% ASD vs. 82% control, *z* = 2.15, *p* < 0.05). No significant group differences emerged in the proportion of participants with valid data for the remaining four TAT images. Overall, the following number of individuals were excluded (range is presented to address different Ns for each TAT image): 11–16 (30–43%) of individuals with ASD, 4–12 (11–32%) of ASD controls, 27–33 (23–31%) of parents of individuals with ASD, and 5–12 (8–24%) of parent controls.

In addition to accounting for track loss, and consistent with prior research (Anderson et al. 2006) each AOI was proportionally expanded by up to 10% based on its original size to create a conservative “buffer,” where when AOIs overlapped fixations were assigned to the AOI with greater social relevance (e.g., faces > bodies) to limit the possibility that errors in gaze detection might contribute to hypothesized group differences in visual attention.

### Analysis Plan

#### Group Differences

*Narrative analyses* For these hypothesis driven analyses, two multivariate analyses of variance (MANOVAs) were conducted for both ASD and control and ASD parent and control parent groups. MANOVAs for individual TAT images were followed with univariate tests if the overall mean LSA for all six images combined was significant. The PB task contained only one outcome variable and was therefore not followed up with univariate comparisons. Eta squared effect sizes are additionally reported (0.01 = small, 0.06 = medium, 0.14 = large).

*Gaze analyses* Given the exploratory nature of gaze analyses for both the PB and TAT task, two-tailed t-tests for each gaze variable (proportion of total fixations and total viewing time to AOIs) were conducted for individuals with ASD, parents, and respective control groups, as well as to examine effects of BAP status. In addition to comparing average performance, each TAT Image (1–6) was examined separately given that images varied in the prominence of AOI and content, and to determine how different stimuli might contribute to overall patterns. Degrees of freedom were corrected to account for non-equality of variances. We additionally adjusted for multiple comparisons using the Benjamini–Hochberg procedure (Benjamini and Hochberg 1995) with a false discovery rate set at 0.1 to account for potentially missing important effects, and report Benjamini–Hochberg corrected *p*-values for all exploratory analyses.

*Relationships between gaze and narrative* Exploratory Pearson correlations were conducted to examine relationships between narrative quality and visual attention metrics within each group. Significance level was set at *p* < 0.05. For exploratory gaze and association analyses, we also report effect sizes using Cohen’s *d* for findings with small to medium or medium to large effect sizes of 0.35 and higher. As in gaze group comparisons, Benjamin-Hochberg adjusted *p*-values are also reported.

*Parent–child correlations* To examine potential familiality of narrative ability in ASD, we applied exploratory Pearson correlations among the parent–child dyads. We examined parent–child correlations for narrative quality only (LSA) for the PB task and for the six TAT images and overall between dyads. Benjamini–Hochberg adjusted *p*-values were also examined to consider false discovery rates, given the exploratory nature of these analyses.

## Results

### Narrative Differences Across Groups and Contexts

**Fig. 2:**
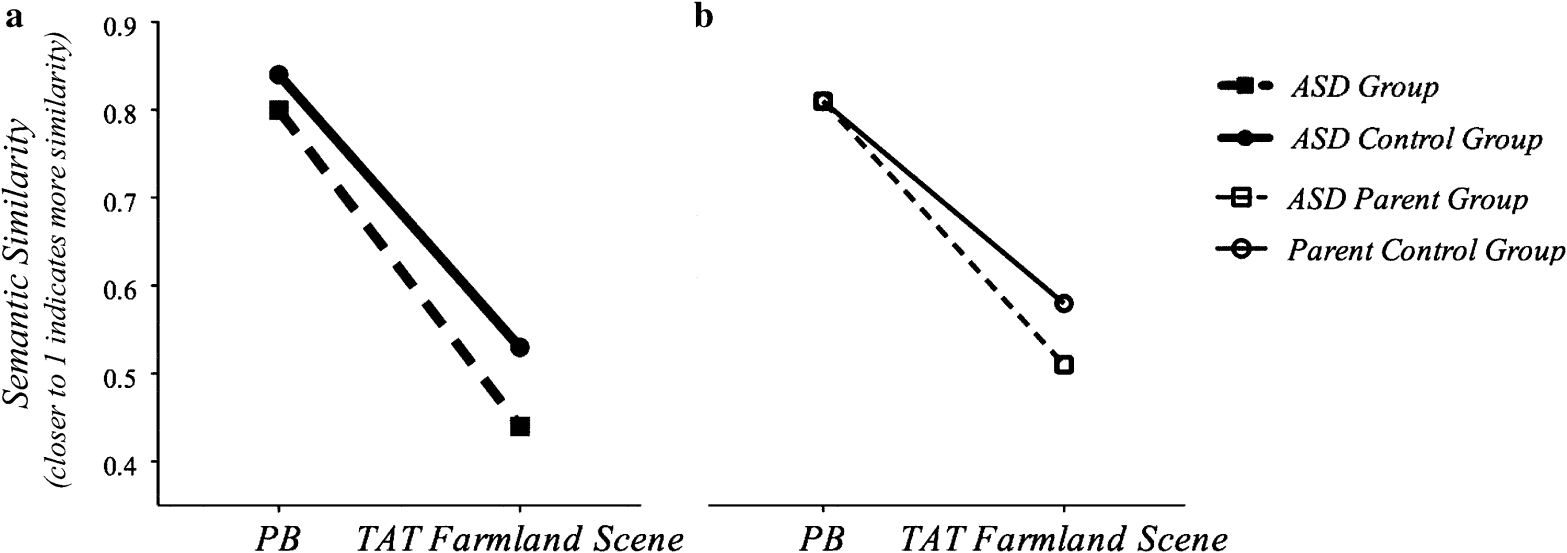
Narrative quality across contexts for ASD and parent groups, indicated by greater LSA scores (i.e., higher semantic similarity) in the structured PB context in both groups, and differences across narrative contexts between **a** individuals with ASD and controls and **b** parents of individuals with ASD and controls

In the PB task, the ASD group demonstrated a small difference from controls (i.e., mean difference = 0.03) of small-to-medium effect in the ASD group F(1,71) = 2.24, *p* = 0.14, *η*^2^ = 0.03), with no differences from controls observed in the ASD parent group F(1,204) = 0.01, *p* = 0.91, *η*^2^ < 0.0001). However, both individuals with ASD and the ASD parent group showed significant differences in narrative quality in the TAT task [F(1,67) = 18.59, *p* < 0.0001, *η*^2^ = 0.22 and F(1,208) = 7.17, *p* < 0.01, *η*^2^ = 0.03), respectively]. Individuals with ASD produced lower quality narratives in the TAT overall, and follow up analyses of individual images showed lower narrative quality in the ASD group in all but one of the six TAT images and in three of the TAT images for parents of individuals with ASD (see Table 3 for group means across narrative tasks). No effects of the BAP were observed. Figure 2 presents narrative results from the PB and an exemplar image in which both individuals with ASD and parents showed differences in the “Farmland” scene (Image 2). Examples of high and low quality narratives from the ASD and parent groups and their respective controls are presented in Tables 4 and 5, respectively.

**Table 3:** Narrative differences across groups

	ASD group, M (SD)	ASD control group, M (SD)	Significance testing, F/*η*^2^	ASD parent group, M (SD)	Parent control group, M (SD)	Significance testing, F/*η*^2^
PB structured narration	0.80 (0.09)	0.83 (0.08)	F(1,71) = 2.24/*0.03*	0.81 (0.12)	0.81 (0.10)	F(1,204) = 0.01/*0.00*
TAT unstructured narration	0.39 (0.11)	0.51 (0.09)	**F(1,67)** = **18.59**/***0.22***	0.45 (0.11)	0.50 (0.08)	**F(1,208)** = **7.17**/***0.03***
1 “Violin Image”	0.42 (0.19)	0.60 (0.09)	**F(1, 67)** = **21.72**/***0.24***	0.51 (0.15)	0.55 (0.16)	F(1,208) = 2.12/*0.01*
2 “Farmland Image”	0.44 (0.16)	0.53 (0.15)	**F(1,67)** = **4.70**/***0.07***	0.51 (0.15)	0.58 (0.11)	**F(1,208)** = **10.26**/***0.05***
3 “Window Image”	0.42 (0.15)	0.44 (0.12)	F(1,67) = 0.04/0.00	0.43 (0.14)	0.46 (0.10)	F(1,208) = 2.03/*0.01*
4 “Surgery Image”	0.37 (0.15)	0.45 (0.14)	**F(1,67)** = **5.01**/***0.07***	0.43 (0.14)	0.47 (0.12)	**F(1,208)** = **4.14**/***0.02***
5 “Sleep Image”	0.35 (0.20)	0.53 (0.14)	**F(1,67)** = **13.93**/***0.17***	0.39 (0.17)	0.43 (0.14)	F(1,208) = 1.60/*0.01*
6 “Man, Woman Gaze Image”	0.35 (0.17)	0.47 (0.18)	**F(1,67)** = **7.73**/***0.10***	0.43 (0.17)	0.48 (0.13)	t(1,208) = 3.48/*0.02*

*η*^2^ convention: 0.01 = small, 0.06 = medium, 0.14 = large effects

M, mean; SD, standard deviation

Bold findings indicate significant differences at the level of *p* < 0.05

**Table 4:** Excerpts of narratives differing in quality from individuals with ASD and ASD controls during the “Farmland Image” of the TAT

Example of strong narrative quality in ASD control	Example of medium quality narrative quality in individuals with ASD
Age = 19.0, sex = female, FSIQ = 118, VIQ = 121, PIQ = 109	Age = 34.9, sex = male, FSIQ = 116, VIQ = 101, PIQ = 131, ADOS severity = 7
Similarity^a^ = 0.70	Similarity^a^ = 0.38
Okay. So there’s a young girl named Jane and she grew up on a farm. Um. The school she goes to is wait she grew up on a farm. And her schoolteacher is very inspiring and encourages really world learning. But her parents just want her to have the lifestyle of a farm girl and just stay in the farm. But Jane really wants to explore the world like her teacher is telling her to. So she’s in the dilemma. As much as she wants to respect her parents she decides to travel and gets the education she wanted.	It looks like it takes place in like the eighteen hundreds. And there’s like a farm with fields. And the guy was telling the woman just that he was going out to work in the fields. And they live on a farm. There was probably corn out there. I think there was a cow in the background.

^a^Narrative quality closer to 1 is higher quality

**Table 5:** Excerpts of narratives differing in quality from parents of individuals with ASD and parent controls during the “Farmland Image” of the TAT

Example of strong narrative quality in parent control	Example of low quality narrative quality in BAP(+) parent
Age = 42.3, sex = female, FSIQ = 118, VIQ = 122, PIQ = 109	Age = 49.8, sex = female, FSIQ = 114, VIQ = 106, PIQ = 121
Similarity^a^ = 0.70	Similarity^a^ = 0.30
Eliza thought back to when she was a child growing up on a farm and she didn’t want to be a farmer’s wife like her mother. So she decided she would teach herself to read because at that time women didn’t go to school. She taught herself to read and because she was such a wonderful scholar she became the first woman in her family and in the state to go to college. And she became a very successful English professor instead of a farmer’s wife. The end.	This is rural England and they’re plowing I don’t really know what kind of story to make up about this. The one girl is wanting to go away and study but she’s supposed to help on the farm. But uh she’s going to be allowed to go and study and I don’t know. She’s Marie Curie and she’ll invent radiation. I don’t know.

^a^Narrative quality closer to 1 is higher quality

#### Gaze Differences Across Groups and Contexts

No differences were observed in visual attention to bodies or faces in the PB task for either the ASD or ASD parent groups. However, the ASD group showed a lower proportion of fixations to setting AOIs than controls (t(59) = − 2.20, *p* < 0.05, *p-adjusted* = 0.50, *d* = − 0.57; mean difference = 2.19%).

**Fig. 3:**
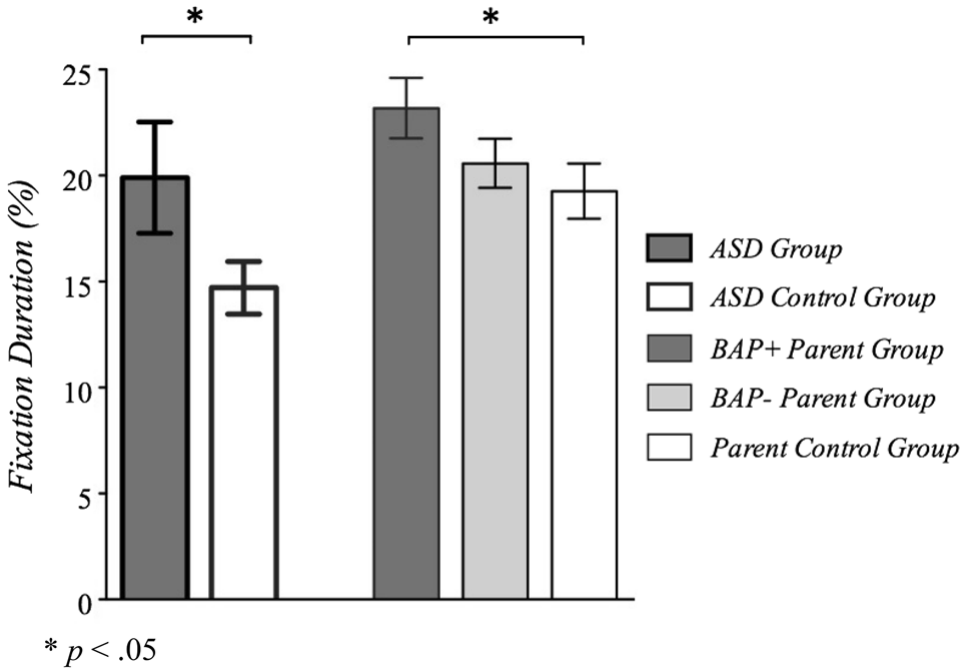
Attention to setting in the Farmland Image (Image 2)

**Fig. 4:**
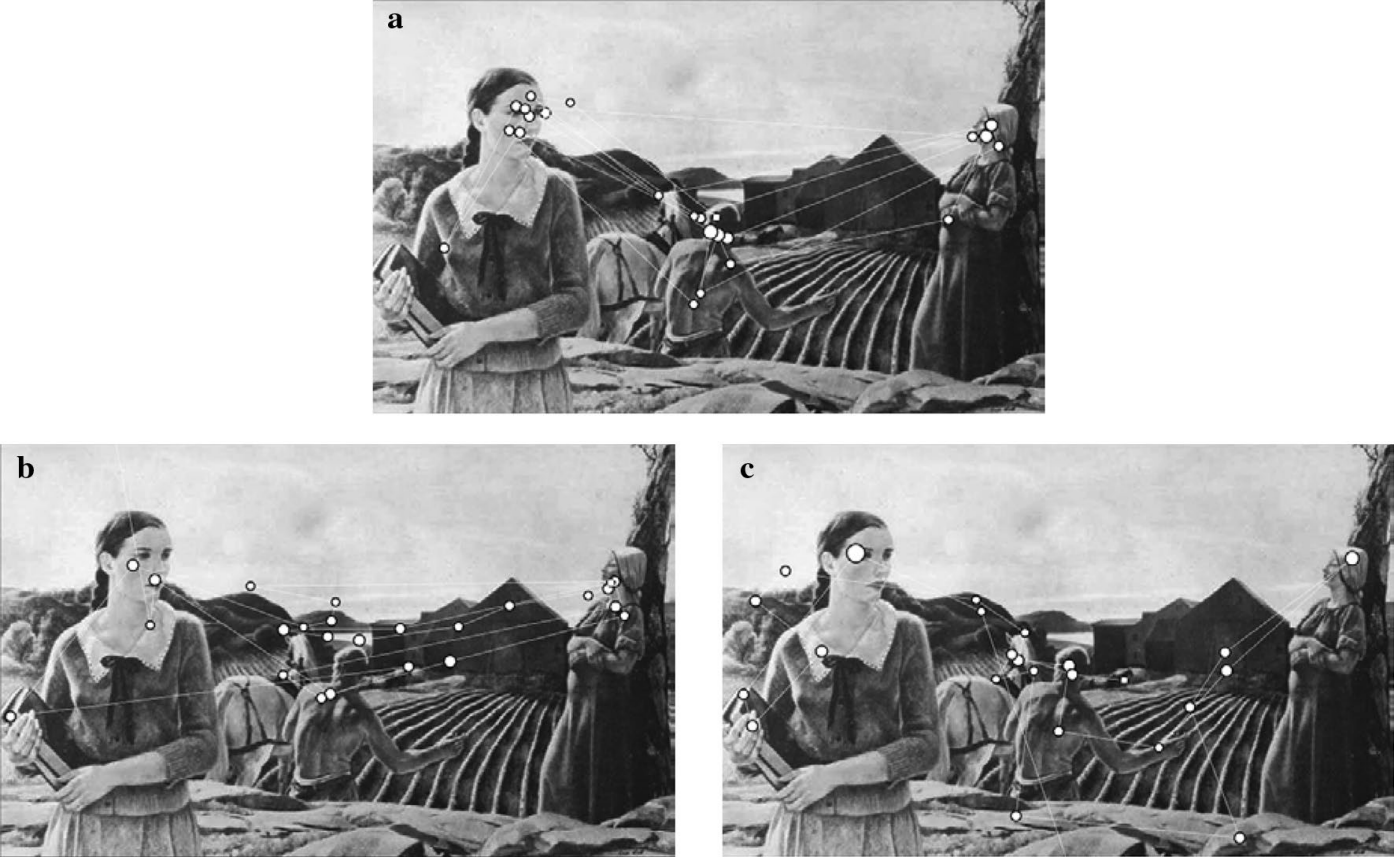
Fixation profiles of **a** typically developing control showing a pattern focused strongly and centrally on animate elements, and their facial regions in particular; **b** ASD (left) and **c** BAP(+) ASD parent (right) gaze paths showing more broadly dispersed gaze paths, focused more on background elements

In the TAT, whereas the ASD group showed several subtle differences from controls, no differences in visual attention were observed between the ASD parent group overall and parent controls. However, the BAP(+) subgroup showed a number of subtle differences from controls and/or BAP(−) parents, as follows. Individuals with ASD and BAP(+) parents showed a medium-effect sized trend toward allocating a greater proportion of visual attention to faces relative to respective control groups, on the images in which faces were featured most prominently [“Man, Woman Gaze”—ASD group proportion fixations t(47) = 1.97, *p* = 0.06, *p-adjusted* = 0.50, *d* = 0.58, mean difference from controls = 8.42%; “Violin”—BAP(+) parents proportion viewing time t(104) = 1.77, *p* = 0.08, *p-adjusted* = 0.67, *d* = 0.34 mean difference from controls = 5.48% and t(111) = 0.1.75, *p* = 0.08, *p-adjusted* = 0.40, *d* = 0.33 mean difference from BAP(−) = 4.79%]. In response to the “Surgery” image, BAP(+) parents attended significantly more to the characters’ faces and less to bodies relative to BAP(−) parents, but not parent controls [faces proportion fixation and viewing time: *t*(105) > − 2.42, *ps* < 0.05, *ps-adjusted* < 0.49, *ds* > − 0.43, mean difference from BAP(−) group = 6.93%], bodies proportion viewing time: t(105) = 2.50, *p* < 0.05, *p-adjusted* = 0.14, *d* = 0.47, mean difference from BAP(−) group = − 5.56%. In response to the image depicting the most complex scene and most highly detailed setting (“Farmland”), both the ASD and BAP(+) groups showed increased attention to the setting relative to controls, though marginally significant in the ASD group [ASD group proportion viewing time, t(34.74) = − 1.79, *p* = 0.08, *p-adjusted* = 0.94, *d* = − 0.49]; BAP(+) parent proportion viewing time, t(108) = − 2.02, *p* < 0.05, *p-adjusted* = 0.67, *d* = − 0.38 (mean differences depicted in Figs. 3, 4).

#### Relationships Between Narrative and Gaze Across Groups and Contexts

No significant associations were detected between visual attention and narrative quality in the PB task for individuals with ASD. For parents of individuals with ASD, decreased attention to the setting of the PB was related to lower narrative quality (for proportion fixations and viewing time *r*s > 0.30, *p*s < 0.002, *adjusted ps* < 0.004).

**Fig. 5:**
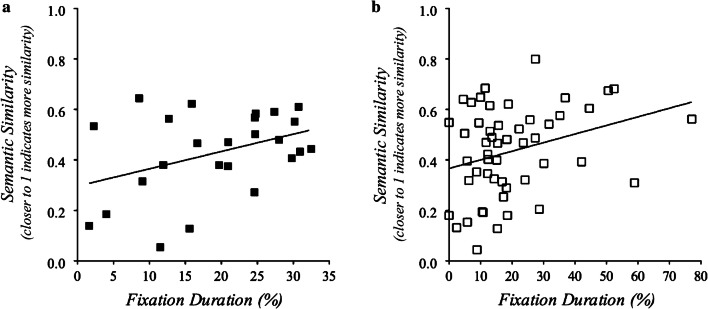
Gaze associations with narrative quality during the TAT. **a** The ASD proband group demonstrated increased fixation duration towards faces and higher LSA scores (i.e., greater narrative coherence, indicated by higher semantic similarity scores) during the Farmland image (Image 2); **b** BAP(+) parent group demonstrated increased fixation duration towards bodies and higher LSA scores during the Man, Woman Gaze image (Image 6)

In the TAT, the ASD group showed significant associations between gaze and narrative quality in response to the “Farmland” image, with lower narrative quality correlated with heightened attention to bodies (proportion viewing time *r* = − 0.41, *p* < 0.05) and a medium association with greater attention to faces (proportion viewing time *r* = 0.39, *p* = 0.051) (Fig. 5a). In the ASD parent group, and particularly the BAP(+) group, fixating more to bodies was associated with *increased* narrative quality in response to one particular TAT image where bodily figures figured prominently (“Man, Woman, Gaze” parent overall *r* = 0.21, *p* < 0.05; BAP(+) *r* = 0.30, *p* < 0.05) (Fig. 5b). Gaze analyses in the TAT did not remain statistically significant at the level of *p* < 0.05 when applying Benjamini Hochberg adjusted *p*-values (*adjusted p-values* ranged from 0.30 to 0.46).

No associations with gaze and narrative were observed in the ASD control group. In the parent control group, better narrative quality was associated with greater attention to faces in two images where faces were prominently featured (“Violin”, proportion duration *r* = 0.29, *p* < 0.*05, adjusted p* = 0.31; *“*Window” proportion duration *r* = 0.38, *p* < 0.*01, adjusted p* = 0.10).

#### Parent–Child Correlations as an Index of Familial Effects of Narrative Ability in ASD families

Analysis of parent–child correlations in narrative ability revealed only one significant association for a TAT image (“Violin” *r* = 0.33, *p* < 0.05, *adjusted p* = 0.21), along with several non-significant correlations in a similar direction in the TAT and PB tasks (*r* ranges: 0–0.33 and 0.11, respectively).

## Discussion

This study investigated narrative skill across contexts in ASD and parents of individuals with ASD. Given well-documented differences in social communication, and narrative in particular, in ASD and evidence of similar, but more subtly expressed differences in parents (Landa et al. 1991, 1992; Losh and Capps 2003; Losh et al. 2008; Loveland et al. 1990; Piven et al. 1997), this study aimed to better characterize narrative across discourse contexts varying in structure and emotional complexity. A primary goal of the study was to determine whether parents might show similar patterns of narrative differences across structured and unstructured contexts that could constitute genetically meaningful phenotypic profiles and provide clues into core skills impacted by genetic liability to ASD. Additionally, we explored visual attention during narration as a potential source of differences in narrative competence, in line with prior work documenting visual attention biases in ASD and among first-degree relatives.

In line with hypotheses related to narrative production, individuals with ASD and parents of individuals with ASD showed parallel patterns of narrative performance, with narrative quality comparable to controls in the highly structured PB context, but with the ASD and ASD parent groups producing less coherent narratives than controls in the less structured, more emotionally evocative TAT narrative task. Gaze differences were also noted in this less structured TAT context, and some associations between gaze and narrative were detected. These findings implicate narrative ability as a complex communication skill that may be impacted by ASD genetic risk. Although differences in visual attention during narrative were subtle and will need to be replicated, evidence of difference in both the ASD and ASD parent groups support the need for further investigation of gaze and language links to understand the origins of the complex social-communicative features associated with ASD.

Consistent with prior literature documenting greater narrative impairments in ASD in less structured contexts (e.g., Diehl et al. 2006; Losh and Capps 2003), individuals with ASD and the ASD parent group showed reduced narrative quality in the less structured TAT narrative task, but did not differ from controls in the highly structured narrative PB task. This pattern could be due to the reduced cognitive and social-emotional demands in the PB task. For example, participants told the PB narrative while viewing a single page at a time, with a clear temporal unfolding of relatively unambiguous events, and characters showing obvious facial expressions of basic emotions. By contrast, the less structured TAT included more ambiguous and emotionally complex scenes, which require understanding of thoughts, emotions, contextual features related to different psychological states (indeed, the TAT was developed with the goal of tapping such complex social perceptual skills) (Murray 1943), and also required narrative generation *after* viewing each image, placing greater cognitive demands (e.g., working memory) on participants. It is perhaps notable that such robust differences in the TAT were evident in spite of explicit instructions to focus on story structure, content, and cognitive/emotional states of characters (i.e., “tell a story with a beginning, middle, and end”, and “discuss thoughts, feelings, and actions of characters”), which would presumably steer individuals’ narratives along somewhat common paths. Although prior studies have not compared narrative ability across different contexts in parents, these findings are consistent with evidence of narrative differences among parents of individuals with ASD when presented with a general direction to tell a story and an initial introductory sentence as a prompt, without any supporting visual stimuli (Landa et al. 1991).

Together, results suggest a relatively specific pattern of narrative differences evident in ASD and among parents, that also showed evidence of familiality in the ASD and ASD parent groups, consistent with a large body of work highlighting subtle differences in social-communication and personality features in first-degree relatives of individuals with ASD thought to reflect genetic liability [i.e., Broad Autism Phenotype; (Piven et al. 1994, 1997; Losh et al. 2008, 2012)]. Familial aggregation of a trait does not necessarily imply a genetic influence [e.g., narrative styles are certainly learned within families, and during parent–child interactions in particular (Haden et al. 1997)]. However, as noted previously, narrative differences were among the first reported phenotypes in early studies documenting the presence of a broad autism phenotype among parents of individuals with ASD (Landa et al. 1991), and considered in this context, the current findings appear to highlight narrative as a fruitful focus for future investigations, such as twin studies, that might more definitively evaluate genetic influence, and the potential of narrative-related skills as ASD endophenotypes.

Evidence of this specific pattern of narrative differences in ASD and parents also builds on prior work applying a computational measure of narrative (i.e., Latent Semantic Analysis, or LSA), showing, importantly, that this method is not only sufficiently sensitive to capture context-dependent narrative deficits in ASD (Lee et al. 2017; Losh and Gordon 2014), but also the more subtle differences evident in clinically unaffected parents. Application of such efficient, automated, and objective computational measures to characterize complex language phenotypes in ASD and among unaffected relatives can provide distinct advantages over hand-coding methods, which, while providing deep characterizations of language samples, are highly labor intensive and difficult to apply to large samples or across different study samples and research groups. The quantitative, continuous index of complex language ability produced by computational methods may also be advantageous for studies of ASD-related endophenotypes, where continuous measures of complex traits, measurable in affected and unaffected individuals can optimize power to detect associations between phenotypes and underlying biological variation. One notable weakness of this computational approach, however, is that more specific aspects of narrative performance (e.g., discussion of character motivations, and mental states shown in prior work to be deficient in ASD) are not captured, which in this study may have impacted our ability to detect associations between gaze patterns and these more specific, and meaningful, aspects of narrative production.

Nonetheless, we did detect subtle differences in visual attention in both the ASD and ASD parent groups. Group comparisons of gaze patterns indicated that, consistent with narrative performance patterns, very few differences were observed during the PB context (individuals with ASD showed a small but statistically significant reduction (i.e., 2%) in fixations to setting elements of PB scenes relative to controls). More differences in gaze were observed during the open-ended TAT task, although the pattern of differences varied across images and were not consistently significant when correcting for multiple comparisons. For example, individuals with ASD and BAP(+) parents attended more to the setting in response to the image from the TAT with the most complexly depicted setting (“Farmland” image). In contrast, individuals with ASD and parents [the BAP(+) group in particular] attended more to faces in response to images from the TAT where facial expressions were prominently featured and the emotional content more ambiguous (e.g., Man, Woman Gaze image). These findings contrast with prior work with different paradigms showing more striking differences in visual attention to social scenes in ASD, including atypical face processing (e.g., see Chita-Tegmark 2016; Frazier et al. 2017; Papagiannopoulou et al. 2014 for reviews). However, the current paradigm was distinct from such prior work in that individuals were *explicitly instructed* to narrate and to discuss the characters’ thoughts and feelings, which likely prompted more focused and directed attentional strategies (and potentially attenuated differences) in social attention than those studied in prior work. Given that successful narration requires attention to both the main characters and the setting that contextualizes characters’ thoughts and actions within a broader theme (Reese et al. 2011), increased allocation of visual attention to the most complex aspects of a given image in the ASD and BAP(+) groups may reflect greater effort to integrate and process visual information to construct meaningful narratives. Therefore, these results may inform future studies examining the role of context in shaping visual attentional differences in individuals with ASD and the BAP.

It is also important to consider that patterns of visual attention where groups differed were not the same aspects of visual attention associated with narrative in either context. Whereas individuals with ASD fixated more intensively on setting in the most visually complex TAT image (“Farmland”), it was an increased attention to bodies that was related to poorer quality narratives. It may be that individuals with ASD and the BAP differ not only in allocation of visual attention, but also in the ways in which they utilize visual information to inform social communication—e.g., even though individuals with ASD and parents with the BAP looked more to faces during emotionally ambiguous TAT images, perhaps they were less able or inclined to capitalize on that information to enrich their narratives. For parent controls, narrative quality increased with greater attention to faces, particularly during images where faces were more prominent, suggesting that they capitalized on this information to inform their narrations. Different associations between narrative quality and attention in the ASD and BAP groups may also indicate that the complexity and degree of ambiguity of a social stimulus impact the attentional strategies employed, with the potential to both miss key aspects of relevant non-social information or to become overly focused on less social stimuli. Additionally, as noted previously, LSA (a global measure of narrative) may not be a sufficiently sensitive index of the finer-grained aspects of narrative that relate to visual attention. Future studies might address these questions by examining gaze and language patterns across different and potentially more sensitive measures of language (ranging from basic language processing skills to more complex language use such as narrative and conversation) and gaze (including moment-to-moment visual attention sequences and synchronized language production), and in larger samples, that could more powerfully index important relationships between gaze and language in real time.

An additional important finding concerns the relative specificity (and subtlety) of gaze differences to the BAP(+) parent group, whereas differences in narrative quality were observed more broadly in the full ASD parent group. Global differences in social communication have been observed among first-degree relatives in other genetically-based disorders impacting language (e.g., specific language impairment; Ruser et al. 2007), raising the possibility that differences observed in the ASD parent group overall reflect more general genetic liability to language disorder, rather than ASD specifically. In line with this possibility, a number of prior family studies of ASD have noted broad-based differences from controls among ASD parent groups, with more specific patterns of differences observed among BAP(+) subgroups, including studies of social cognition (Losh et al. 2009), face processing (Adolphs et al. 2008; Yucel et al. 2015), and visual attention during a language processing task (Nayar et al. 2018). Future work including comparison groups of parents of children with other genetically-based language disorders will be informative in teasing out ASD-specific risk markers evident in parents. It could also be the case that more detailed characterization of narrative ability (rather than the global narrative analysis examined in this study) could reveal patterns of narrative differences more specific to parents with the BAP.

In summary, results from this study highlight a specific pattern of differences in narrative skill in individuals with ASD and among parents (particularly those with the BAP), that may be linked with visual attention patterns, where differences are most robustly observed in unstructured contexts involving emotionally evocative, ambiguous scenes. Such overlapping phenotypic patterns in ASD and among parents suggest that narrative ability and related visual attention patterns may be important phenotypes that could be used in future studies indexing genetic liability to ASD, which could help to inform the basis of the complex social-communicative impairments in ASD. Findings that many, but not all, differences among parents were driven by the BAP(+) subgroup (e.g., with all parents showing differences in narrative, yet most gaze differences were specific to the BAP) may also have important implications for understanding mechanistic differences relating to core language-related phenotypes in ASD and the BAP.

## Abbreviations

PB: : Picture book task
TAT: : Thematic Apperception Test
